# Suizidprävention im Hamburger Strafvollzug – Analyse der Fälle 2013–2022 und Auswertung von Interviews mit Inhaftierten und Mitarbeitenden

**DOI:** 10.1007/s00103-025-04154-x

**Published:** 2025-11-12

**Authors:** Sabrina Kunze, Celine Nguyen, Lena Harms, Carlotta Herrmann, Nadine Ritter, Klaus Püschel, Peer Briken, Benjamin Ondruschka

**Affiliations:** 1 Institut für Rechtsmedizin, Universitätsklinikum Hamburg-Eppendorf, Butenfeld 34, 22529 Hamburg, Deutschland; 2 Deutsches Zentrum für Suchtfragen des Kindes- und Jugendalters (DZSKJ), Universitätsklinikum Hamburg-Eppendorf, Hamburg, Deutschland; 3 Institut für Sexualforschung, Sexualmedizin und Forensische Psychiatrie, Universitätsklinikum Hamburg-Eppendorf, Hamburg, Deutschland

**Keywords:** Untersuchungshaftanstalt, Suizidprävention, Risikofaktoren, Aktenanalyse, Leitfadeninterview, Pre-trial detention center, Suicide prevention, Risk factors, File analysis, Semi-structured interview

## Abstract

**Hintergrund:**

Seit 1962 werden Suizidfälle im Hamburger Strafvollzug wissenschaftlich hinsichtlich Ursachen und Risikofaktoren analysiert, um Maßnahmen der Suizidprävention zu verbessern. Die vorliegende Arbeit knüpft daran an und untersucht die Suizide von 2013–2022. Ergänzend geben Interviews mit Inhaftierten und Mitarbeitenden Einblicke in Herausforderungen der Suizidprävention.

**Methoden:**

Die Studie folgte einem Mixed-Methods-Ansatz. Für die Suizidfälle 2013–2022 wurden rechtsmedizinische Sektionsprotokolle sowie Gefangenen-Personalakten und -Gesundheitsakten quantitativ-deskriptiv ausgewertet. Zusätzlich wurden im Zeitraum September bis Oktober 2022 qualitative Leitfadeninterviews mit Mitarbeitenden und Inhaftierten der Untersuchungshaftanstalt zu ihren Erfahrungen und dem Umgang mit Suizid geführt. Die Auswertung basierte auf einer qualitativen Inhaltsanalyse nach deduktiv-induktiver Kategorienbildung.

**Ergebnisse:**

Im Erhebungszeitraum wurden 20 Suizide verzeichnet, wobei die Prävalenz in den Jahren 2017, 2020 und 2021 am höchsten war (je 4 Fälle). Ein Zusammenhang mit der Pandemie lässt sich nicht belegen. 12 Suizide (60 %) ereigneten sich in Untersuchungshaft, 18 (90 %) betrafen Männer. Häufigste Suizidmethode war die Strangulation (18 Fälle, 90 %). Die Interviews weisen auf potenziell suizidrelevante Aspekte der Arbeits- und Haftbedingungen hin.

**Diskussion:**

Im Vergleich zu den früheren Erhebungen ist die jährliche Anzahl von Suizident:innen leicht gesunken. Die Untersuchung zeigt, dass Suizide im (Hamburger) Strafvollzug eine persistierende Herausforderung darstellen. Präventive Maßnahmen wie Suizidscreenings, psychologische Betreuung, Fort- und Weiterbildungen für Personal sowie strukturelle und bauliche Anpassungen können zur Risikominimierung beitragen.

**Zusatzmaterial online:**

Zusätzliche Informationen sind in der Online-Version dieses Artikels (10.1007/s00103-025-04154-x) enthalten.

## Abstract (en)

**Background:**

Since 1962, suicide cases in Hamburg′s penal system have been scientifically analyzed with regard to causes and risk factors in order to improve suicide prevention measures. The present study builds on this long-term research tradition and examines suicides occurring between 2013 and 2022. Additionally, interviews with inmates and staff provide insights into the challenges of suicide prevention in custody.

**Methods:**

The study followed a mixed-methods approach. For suicide cases from 2013 to 2022, forensic autopsy reports as well as inmate personnel and health records were quantitatively and descriptively analyzed. Additionally, between September and October 2022, qualitative guideline-based interviews were conducted with staff and inmates of the pre-trial detention center regarding their experiences and approaches to suicide. The qualitative data were analyzed using content analysis based on a deductive–inductive category system.

**Results:**

During the study period, 20 suicides were recorded, with the highest prevalences observed in 2017, 2020, and 2021 (four cases each). No direct link to the pandemic could be established. Twelve suicides (60%) occurred in pre-trial detention, and 18 (90%) involved men. The most common method was strangulation (18 cases, 90%). The interviews indicate potentially suicide-relevant aspects of working and detention conditions.

**Discussion:**

Compared to earlier surveys, the annual number of suicides has slightly decreased. The findings indicate that suicides in the (Hamburg) penal system remain a persistent challenge. Preventive measures such as suicide screenings, psychological support, training, and further education for staff, as well as structural and architectural adjustments, may reduce risk factors for suicide.

## Hintergrund

Ein Suizid stellt neben Erkrankungen eine der häufigsten Todesursachen im deutschen Justizvollzug dar. Dies verdeutlicht die Notwendigkeit gezielter Präventionsmaßnahmen. Bundesweite Analysen zeigen seit 2013 einen Anstieg der Suizidrate von 74,5 (2013) auf 159,1 (2021) pro 100.000 Inhaftierte, mit Ausnahme der Jahre 2018 und 2019 [1]. Die Studie von Meischner-Al-Mousawi et al. [1] stellte zudem einen Anstieg der Suizide während der Pandemie (2020 bis 2022) fest.

In Hamburg stieg nach Angaben der Behörde für Justiz und Verbraucherschutz der Freien und Hansestadt Hamburg (BJV) die Anzahl psychischer Erkrankungen bei Inhaftierten von 384 Fällen im Jahr 2022 auf 681 im Jahr 2024 an. Hierbei kann nicht abschließend geklärt werden, ob der Anstieg auf eine erhöhte Diagnostik und Therapieinanspruchnahme zurückzuführen ist. Gleichwohl erfordern die steigenden Prävalenzen eine adäquate Anpassung der Versorgungsstrukturen für Inhaftierte.

**Abb. 1:**
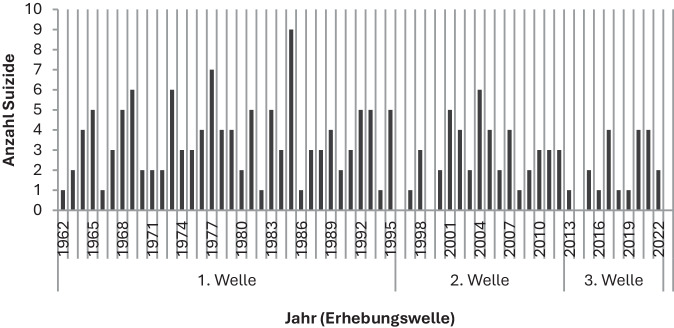
Anzahl der Suizide in Hamburger Strafvollzugsanstalten in den 3 Erhebungszeiträumen 1962–1995, 1996–2012 und 2013–2022

Seit über 60 Jahren werden Suizidfälle im Hamburger Strafvollzug wissenschaftlich erfasst und hinsichtlich ihrer Ursachen und Risikofaktoren analysiert, um Maßnahmen der Suizidprävention fortlaufend zu verbessern. Von 1962–2022 wurden insgesamt 190 Suizide dokumentiert (Abb. 1; [2–5]). Die Auswertungen 1962–1995 erfolgten durch Granzow und Püschel (1. Erhebungszeitraum), jene für 1996–2012 durch Petersen et al. (2. Erhebungszeitraum). Die vorliegende Arbeit untersucht die Jahre 2013–2022 als 3. Erhebungszeitraum. Insgesamt wiesen die Hamburger Vollzugsanstalten stärkere Schwankungen in den Suizidfällen auf als Vollzugsanstalten im Bundesdurchschnitt (Tab. 1). Während des Beginns der Pandemie (2020/2021) traten in Hamburg Prävalenzspitzen auf, wohingegen die Suizidraten in der Allgemeinbevölkerung relativ konstant blieben.

**Abb. 2:**
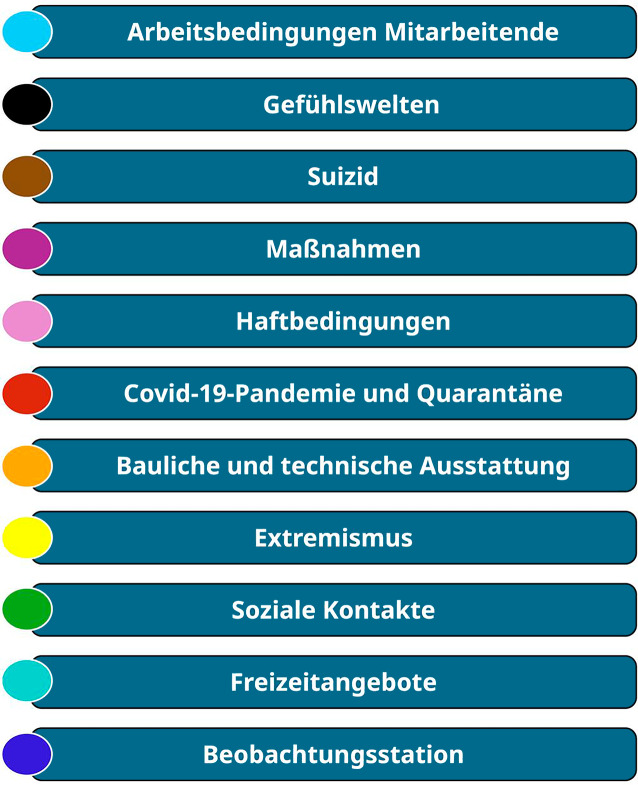
Kategoriensystem der Leitfadeninterviews

**Tab. 1:** Suizidraten in Haft und der Allgemeinbevölkerung

Jahr	Suizidrate in Haft, deutschlandweit (Anzahl pro 100.000 Inhaftierte)	Suizidrate in Haft, hamburgweit (Anzahl pro 100.000 Inhaftierte)	Suizidrate Allgemeinbevölkerung, hamburgweit (Anzahl pro 100.000 Einwohnende)
2013	74,5	77,1	13,5
2014	83,7	Keine Suizide	14,4
2015	105,3	173,6	14,3
2016	119,6	84,5	15,9
2017	127,7	324,4	13,9
2018	94,9	76,1	13,7
2019	65,4	76,6	15,1
2020	129,5	335,9	13,9
2021	159,1	316,7	11,3
2022	141,4	166,9	13,7

Datenquelle: Statistisches Amt für Hamburg und Schleswig-Holstein, Strafvollzugsstatistik 2022, letzter Zugriff: 30.07.2025, 21:20 Uhr

In der vorliegenden Arbeit werden für die Suizidfälle im Zeitraum 2013–2022 soziodemografische, gesundheits- und haftbezogene Daten sowie Daten zum Suizidgeschehen ausgewertet. Mithilfe von leitfadengestützten Interviews mit Inhaftierten und Mitarbeitenden werden die Ursachen und Risikofaktoren für Suizide untersucht. Ziel der Arbeit ist es, Maßnahmen zur Verbesserung der Suizidprävention unter Einbezug aktueller Einflüsse, bspw. durch die SARS-CoV-2-Pandemie, abzuleiten.

## Methoden

Das Studiendesign folgt einem Mixed-Methods-Ansatz, bestehend aus einer quantitativen Aktenanalyse. Von den Suizidfällen im Zeitraum 2013–2022 wurden rechtsmedizinische Sektionsprotokolle, Gesundheitsakten und Gefangenen-Personalakten untersucht. Zusätzlich fanden 20 qualitative Leitfadeninterviews mit 10 Inhaftierten sowie 10 Mitarbeitenden der Hamburger Untersuchungshaftanstalt (UHA) statt. Die Interviews zielten darauf ab, aktuelle Herausforderungen im Hinblick auf selbstschädigendes Verhalten möglichst differenziert zu identifizieren und aktuelle Bedürfnisse zu erfassen. Für die quantitative Auswertung wurde ein 148 Items umfassender Katalog zu biografischen, psychologischen, infektiologischen und deliktbezogenen Aspekten in Form eines Kategoriensystems erstellt. Weitere Informationen zu den wesentlichen Items sind im Onlinematerial, Anhang C zu finden. Aufgrund der geringen Fallzahl erfolgte eine rein deskriptive Darstellung der Ergebnisse (absolute Zahlen und Prozentangaben) ohne inferenzstatistische Verfahren. Die Analyse erfolgte mit IBM SPSS Statistics (Version 27).

Die qualitativen Interviews basierten auf je 9 Leitfragen mit Folgefragen (siehe Onlinematerial, Anhänge A und B). Inhaftierte erhielten im Anschluss daran ein Guthaben von 5 €. Die qualitative Auswertung erfolgte in Form einer Inhaltsanalyse [6] unter Verwendung einer deduktiv-induktiven Kategorisierung mit MAXQDA 2022 (VERBI Software, 2021). In der deduktiven Phase werden Kategorien entwickelt, die auf theoretischen Annahmen sowie der Struktur des Interviewbogens basieren und dann auf das Datenmaterial angewendet werden. In der induktiven Phase werden Kategorien gebildet, die direkt während der Analyse aus den Daten selbst hervorgehen. Intercoder-Reliabilität wurde durch mehrfaches Kodieren und Überarbeitung des Schemas sichergestellt.

Bei der Auswahl der Inhaftierten als Interviewpartner für die Leitfadeninterviews sollte auf eine möglichst große Streuung von Merkmalen geachtet werden, wie sie für die Haftpopulation in Deutschland typisch ist. Entsprechend wurde eine Stichprobe ausschließlich männlicher Gefangener angestrebt, da deren Anteil in Deutschland bei ca. 95 % liegt [7]. Die Altersverteilung der vorliegenden Stichprobe konstituierte sich wie folgt: jeweils 4 Personen im Alter von 18–25 Jahren und 26–45 Jahren sowie 2 Personen zwischen 46 und 70 Jahren. Hinsichtlich der ethnischen Zugehörigkeit sollten die Teilnehmenden unterschiedliche Migrationshintergründe aufweisen, wobei ausreichende Deutschkenntnisse Voraussetzung für die Teilnahme waren (3–4 Personen mit Migrationshintergrund). Weitere 6–7 Personen waren deutsche Staatsbürger ohne Migrationshintergrund. Hinsichtlich der Dauer der Inhaftierung sollten Personen mit kurzen Haftstrafen (≤ 3 Monate, 6 Personen) und Personen mit langen Haftstrafen (> 3 Monate, 4 Personen) interviewt werden. Die Deliktstruktur wurde in 2 Bereiche unterteilt: Deliktbereich I: Diebstahl (4 Personen), und Deliktbereich II: Mord/Totschlag, Sexualdelikte, Körperverletzung/Raub (6 Personen). Die Interviewpartner sollten weiter unterteilt werden in: Ersttäter (7 Personen) und Wiederholungstäter (3 Personen).

Die Mitarbeitenden-Stichprobe bestand ebenfalls aus 10 Personen (1 Beamtin, 1 weibliche Pflegekraft, 8 Männer aus unterschiedlichen Berufsgruppen: u. a. Psychologen, Ärzte, Abteilungsleiter). 5 Personen waren 18–43 Jahre alt, weitere 5 waren 44–67 Jahre alt. 3–4 Personen hatten bis zu 5 Jahre, 6–7 mehr als 5 Jahre Berufserfahrung; alle sollten idealerweise Erfahrung mit suizidalen Inhaftierten haben. Die Rekrutierung erfolgte durch das UHA-Personal über persönliche Ansprache und Flyer.

5 weibliche Interviewerinnen (1 Kriminologin, 3 Studierende der Psychologie, 1 Studierende der Kriminologie) führten die Interviews durch. Die Studierenden wurden im Vorfeld geschult. Des Weiteren wurde jedes Interview im Tandem geführt – eine Person moderierte, die andere dokumentierte. Die Interviews wurden aufgezeichnet und später transkribiert.

## Ergebnisse

Im Folgenden werden Ergebnisse der quantitativen und qualitativen Auswertung präsentiert.

### Quantitative Auswertung der Suizidfälle 2013–2022 (Aktenanalyse)

Zunächst werden die Ergebnisse der quantitativen Auswertung dargestellt. Von den 20 Suizidfällen im Zeitraum 2013–2022 wurden, soweit vorhanden, rechtsmedizinische Sektionsprotokolle (20), Gesundheitsakten (18) und Gefangenen-Personalakten (19) untersucht.

#### Soziodemografische Daten.

Die Verteilung der soziodemografischen Merkmale der 20 Suizident:innen und die Auflistung der Suizide im Verhältnis zu den Strafgefangenen und Sicherheitsverwahrten werden in Tab. 1 und 2 ersichtlich.

**Tab. 2:** Soziodemografische Daten zu den 20 Suizidfällen im Hamburger Strafvollzug (2013–2022)

Merkmal (Anzahl vorhandener Daten)	Ausprägung	Anzahl *n* (%)
Geschlecht (20)	Männlich	18 (90 %)
	Weiblich	2 (10 %; davon 1 transgender)
Alter (20) [Altersspanne: 21–56 Jahre, M = 36,6]	21–25 Jahre	2 (10 %)
	26–30 Jahre	5 (25 %)
	31–35 Jahre	4 (20 %)
	36–40 Jahre	3 (15 %)
	41–45 Jahre	1 (5 %)
	46–50 Jahre	2 (10 %)
	51–55 Jahre	2 (10 %)
	55–60 Jahre	1 (5 %)
Staatsangehörigkeit (20)	Deutsch	8 (40 %)
	Polnisch	5 (20 %)
	Afghanisch	2 (10 %)
	Eritreisch	1 (5 %)
	Estnisch	1 (5 %)
	Georgisch	1 (5 %)
	Guinea-bissauisch	1 (5 %)
	Kosovarisch	1 (5 %)
	Syrisch	1 (5 %)
Familienstand (19)	Ledig/alleinlebend	11 (58 %)
	In Beziehung	6 (32 %)
Fester Wohnsitz vor Inhaftierung (18)	Ohne festen Wohnsitz	10 (56 %)
	Mit festem Wohnsitz	8 (44 %)
Schulabschluss (8)	Abitur	2 (25 %)
	Hauptschulabschluss	2 (25 %)
	Realschulabschluss	2 (25 %)
	Kein Schulabschluss	2 (25 %)
Berufsqualifikation (15)	Abgeschlossene Berufsausbildung	6 (40 %)
	Anlernberuf	6 (40 %)
	Ohne Berufsabschluss	3 (20 %)

#### Gesundheitsdaten.

3 Suizident:innen (15 %) hatten vor ihrem Tod über chronische Schmerzen berichtet, je eine Person (5 %) über eine Tic-Störung oder Atemprobleme. Zudem lagen jeweils bei einer Person (5 %) chronische Bronchitis, Atheromatose, Hepatitis, chronische Gastritis oder ein Bandscheibenvorfall vor. 2 Personen (10 %) litten unter Tremor.

Bei 18 Suizident:innen (90 %) wurde während der Haft eine psychiatrische Abklärung durchgeführt; in 2 Fällen fehlten entsprechende Angaben. 15 Personen (75 %) wurden im letzten Querschnittsbefund als „nicht“ oder „eher nicht suizidgefährdet“ eingeschätzt. Bei 16 Personen (80 %) wurde eine psychische Erkrankung dokumentiert, bei den anderen 4 Fällen (20 %) fehlten die entsprechenden Angaben. Jeweils 5 Personen (25 %) litten an einer Alkohol- oder Substanzkonsumstörung, 3 (15 %) an einer psychotischen Störung oder Depression, 2 (10 %) an einer Persönlichkeitsstörung und je eine (5 %) an Geschlechtsinkongruenz oder einer Angststörung.

#### Haftbezogene Daten.

Zum Zeitpunkt des Suizids befanden sich 12 Suizident:innen (60 %) in Untersuchungshaft, 6 (30 %) in Strafhaft, je eine Person (5 %) in Abschiebehaft bzw. im Überstellungsverfahren aus einem anderen Bundesland. Weiterhin waren 14 Personen (70 %) bereits mindestens einmal inhaftiert, 7 (35 %) davon mehr als 2‑mal.

Die Anlassdelikte umfassten: Betrug (1), Verstöße gegen das Betäubungsmittelgesetz (4), Gewaltdelikte (9), vorsätzliche Tötungsdelikte, inkl. Versuch (4), ein politisch rechts motiviertes Delikt (1) und ein Fall von Mitgliedschaft in einer terroristischen Organisation im Ausland (1).

#### Daten zum Suizidgeschehen.

Von den untersuchten Suiziden fanden 13 (65 %) in der UHA statt. Der zur Einschätzung der Suizidalität neu zugeführter Inhaftierter der UHA vorgesehene Suizid-screeningbogen wurde nicht in jedem Fall angewendet. Auch in den einzelnen Strafvollzugsanstalten Hamburgs nutzten geschulte Vollzugsbeamt:innen nicht immer einen Suizidscreeningbogen, sondern stellten einzelne Fragen zur Suizidabklärung. Bei den insgesamt 20 Suiziden zwischen 2013 und 2022 wurde der Suizidscreeningfragebogen in *n* = 15 der Fälle (75 %) eingesetzt. In einem Fall trat der Suizid innerhalb des angestrebten Zeitrahmens zur Suizidabklärung nach Zuführung und vor der Verwendung des Suizidscreeningbogens ein. 3 Personen (15 %) wurden aus anderen Gefängnissen transferiert, bei diesen erfolgte kein Suizidscreening, eine Person wurde aus dem Maßregelvollzug verlegt. In 2 Fällen wurden anstelle des Suizidscreeningbogens 2 Fragen zur aktuellen Suizidalität gestellt.

Im Berichtszeitraum stellen die Jahre 2017, 2020 und 2021 Prävalenzspitzen mit jeweils 4 Suiziden (20 %) dar. Hinsichtlich des Zeitpunkts der Entdeckung der suizidierten Personen wurden 11 Fälle (55 %) in den frühen Morgenstunden zwischen 6 und 7 Uhr (Zeitpunkt des Aufschlusses) festgestellt. Jeweils 4 Fälle (20 %) wurden zwischen 7–16 Uhr bzw. zwischen 16 und 17 Uhr entdeckt. Ein weiterer Suizid (5 %) wurde zwischen 22 und 23 Uhr festgestellt. Am häufigsten wurde bei der Obduktion Strangulation (Erhängen, Erdrosseln) als Todesursache festgestellt (18 Fälle, 90 %).

### Qualitative Auswertung der Inhaftierten- und Mitarbeitendeninterviews

Aus den 20 Interviews wurde ein deduktiv-induktives Kategoriensystem (Abb. 2) entwickelt, aus welchem exemplarisch 4 Kategorien beschrieben werden, die suizidpräventive Bedarfe aufzeigen sollen. Ergänzend werden Zitate zu subjektiven Empfindungen der interviewten Personen eingefügt, um einen vertieften Einblick in deren Erlebniswelt zu ermöglichen. Dabei wird kein Anspruch auf Allgemeingültigkeit oder objektiven Wahrheitsgehalt erhoben.

#### Arbeitsbedingungen Mitarbeitende.

Mitarbeitende und Inhaftierte berichteten gleichermaßen von zwischenmenschlichen Schwierigkeiten, insbesondere aufgrund von Sprachbarrieren. Laut Aussagen der Mitarbeitenden findet die psychiatrische Basisversorgung in der UHA nur an 3 Werktagen durch Belegpersonal statt. Fortwährender Personalmangel und Schichtarbeit erschweren die Teilnahme an Fortbildungen, obwohl diese angeboten werden und bekannt sind. Die hierfür erforderliche Eigeninitiative, etwa durch Schicht- oder Urlaubswechsel, wurde von den Mitarbeitenden als zusätzliche Hürde wahrgenommen. Zudem lehnen viele Mitarbeitende nach belastenden Ereignissen wie Suiziden unterstützende Angebote (z. B. durch kirchliche Einrichtungen oder den Psychologischen Dienst) ab – aus Sorge, dadurch weniger kompetent und professionell zu wirken. Emotionale Belastungen werden laut Aussagen der Mitarbeitenden meist nur im Kolleg:innenkreis oder privaten Umfeld angesprochen. Zudem wurde von Vermeidungsstrategien wie Verdrängung oder Grübeln, insbesondere in den ersten Tagen nach einem Suizid, berichtet.

#### Zur Beobachtungsstation.

In Hamburg werden Inhaftierte bei suizidalen Absichten in den besonders gesicherten Haftraum (bgH) oder auf die Sicherungs- und Beobachtungsstation (SuBs) verlegt. Im bgH erfolgt die Unterbringung stehend oder auf dem Boden sitzend, da in diesem keine Sitzmöbel vorhanden sind. Einige Inhaftierte äußerten, ihren Leidensdruck verschwiegen zu haben, um nicht in den bgH verlegt zu werden. Diese Hafträume wurden in den Interviews von den Inhaftierten als sehr negativ beschrieben, z. B.:

„Also wenn Sie dann nachts auf den Knopf gehen und sagen ich kann nicht mehr, ich dreh hier durch, ich raste aus, dann kommen Sie höchstens wieder in A2 unter Beobachtung mit Licht an, wo Sie nicht schlafen können, also das ist schon sehr, ja das ist schon ne krasse Erfahrung, sehr sehr sehr demütigend“ (U1_2022, Pos. 30).

#### Freizeitangebote.

Die Inhaftierten berichteten in den Interviews, dass kein ausreichendes Angebot an Arbeits‑, Freizeit‑, Sportangeboten sowie Sprachkursen vorhanden ist, um für alle Interessierten die regelmäßige Teilhabe an Angeboten zu ermöglichen. Offene Stationen, Umschluss (zeitlich begrenzte Option, sich gegenseitig in einem Haftraum zu besuchen und sich gemeinsam einschließen zu lassen) und Freizeitgruppen seien einige der wichtigsten Bestandteile des Lebens in der UHA, da diese an den Alltag außerhalb des Justizvollzugs erinnern würden. Partiell mussten Inhaftierte, u. a. abhängig vom Haftstatut, mehrere Monate warten, um eines der Angebote wahrnehmen zu können.

#### SARS-CoV-2-Pandemie und Quarantäne.

In den Jahren 2020 und 2021, die sehr stark durch Einflüsse der SARS-CoV-2-Pandemie geprägt waren, ereigneten sich in den Hamburger Gefängnissen jeweils 4 Suizide. Obgleich ein kausaler Zusammenhang zwischen pandemiebedingten Einflüssen und einem erhöhten Suizidrisiko im Rahmen dieser Untersuchung nicht abschließend festgestellt werden kann, berichteten sowohl Mitarbeitende als auch Inhaftierte von negativ wahrgenommenen Auswirkungen der strengeren Regularien. Für die Inhaftierten gab es in diesem Zeitraum Einschränkungen durch Quarantänemaßnahmen, insbesondere den Verlust von sozialen und religiösen Kontakten sowie den Wegfall von Freizeitaktivitäten. Während der Höhepunkte der Pandemie war die zusätzliche Belastung durch Quarantänemaßnahmen und lang anhaltende Personalausfälle für die Mitarbeitenden besonders hoch. Das Tragen medizinischer Masken wurde von den Inhaftierten und von vielen Mitarbeitenden nicht als störend empfunden. Die Quarantänezeit selbst wurde von beiden Gruppen als sehr belastend beschrieben, da über mehrere Wochen hinweg teilweise keine Möglichkeit zum Einkaufen, Duschen oder zur Wahrnehmung von gewohnten Freistunden bestand. Eine in der UHA tätige Person äußerte sich so:

„Und am Anfang der Corona-Pandemie hatten wir’s relativ häufig, dass eben eine ganze Station unter Verschluss genommen wurde, wenn auch nur einer infiziert war. War natürlich Mist, da war die, wie heißt das, Quarantänezeit ja auch noch länger“ (M2_2022, Pos. 103).

## Diskussion

Inhaftierte haben ein deutlich erhöhtes Suizidrisiko, insbesondere in der Untersuchungshaftanstalt (UHA; [8, 9]). Daraus ergibt sich die Notwendigkeit, Risikofaktoren frühzeitig zu erkennen, gezielte Maßnahmen umzusetzen und kontextuelle Herausforderungen zu berücksichtigen. Zur besseren Prävention sollen künftig standardisierte Screening-Verfahren in allen Hamburger Haftanstalten etabliert, die psychiatrisch-psychotherapeutische Präsenz in der UHA gestärkt und der Zugang zu Fortbildungen für Mitarbeitende erleichtert werden [10, 11]. Darüber hinaus sollten anstelle von bgH Suizidpräventionsräume eingerichtet werden und die Zeit in Isolationshaft weitestgehend reduziert werden [12, 13]. Zudem sollte ein Quarantänekonzept für potenzielle Ausnahmesituationen erstellt werden, welches die Bereitstellung von Gruppen- und Freizeitaktivitäten fokussiert.

### Vergleich mit den vorherigen Erhebungszeiträumen

Die jährliche Anzahl der verwirklichten Suizide in den Hamburger Haftanstalten hat im Vergleich zu den vorherigen beiden Erhebungszeiträumen absolut und relativ abgenommen (1. Erhebungszeitraum: 3,5 pro Jahr pro 100.000 Inhaftierte, 2. Erhebungszeitraum: 2,9 pro Jahr, aktuell: 2,0 pro Jahr).

Im Rahmen der vorliegenden Erhebung konnten verschiedene Risikofaktoren deskriptiv erfasst werden. Die Ergebnisse können mit den vorangegangenen Analysen verglichen werden.

Hinsichtlich des Alters der Suizident:innen zeigte sich sowohl in der vorliegenden Untersuchung als auch in Erhebungszeitraum 1, dass die Altersgruppe der 26- bis 30-Jährigen am häufigsten betroffen war, wohingegen im 2. Erhebungszeitraum die meisten Suizident:innen zwischen 41 und 45 Jahren alt waren.

Das Verhältnis der Nationalitäten über die 3 Untersuchungszeiträume veränderte sich stetig. Während im 1. und 2. Erhebungszeitraum 88 % bzw. 54 % der Suizident:innen deutsche Staatsangehörige waren, sank die Anzahl in der aktuellen Studie auf 40 %. Dies könnte einerseits mit der wachsenden Zuwanderung ausländischer Personen assoziiert werden [14], andererseits könnte es steigende xenophobe Tendenzen in der Gesellschaft [15–18] und der Strafverfolgung aufzeigen, durch welche ausländisch gelesene Personen häufiger strafrechtliche Konsequenzen erfahren [19–21]. Im 2. Erhebungszeitraum waren vor der Inhaftierung 22 % der Personen arbeitslos, während im aktuellen Zeitraum dieser Anteil auf 45 % anstieg. Dies erscheint zunächst in Anbetracht der vorliegenden Abwärtstendenz der Arbeitslosenquote in Hamburg kontraintuitiv [22], lässt sich jedoch durch einen international erkennbaren Trend erklären, der einen Zusammenhang zwischen Arbeitslosigkeit und erhöhtem Suizidrisiko aufzeigt [23].

Im aktuellen Zeitraum fanden 65 % der Suizide in der UHA statt, im 2. Erhebungszeitraum waren es 58 % und im 1. Erhebungszeitraum 52 %. Dies bestätigt eine höhere Suizidwahrscheinlichkeit in der UHA im Vergleich zum restlichen Strafvollzug, wie bereits seit vielen Dekaden auf internationaler Ebene bekannt ist [12]. Abweichungen lassen sich auf die niedrige Fallzahl im derzeitigen Berichtszeitraum zurückführen. Ferner war in den 3 Erhebungszeiträumen das Erhängen die mit Abstand (84–90 %) am häufigsten gewählte Suizidmethode, meistens mithilfe von Bettlaken und Kleidungsstücken durchgeführt.

### Einschätzung von Suizidalität und Umgang mit selbstschädigenden Verhaltensweisen

Bereits in der Vergangenheit haben diverse Untersuchungen ein erhöhtes Suizidrisiko in den ersten 48 h nach Aufnahme in der UHA gezeigt [24, 25], wodurch die Relevanz zeitnaher und valider Suizidscreenings in diesem Zeitraum essenziell ist. In Anbetracht des aktuellen Forschungsstands wäre der derzeit reliabelste Weg zur Erkennung akuter suizidaler Zustände eine Kombination aus dem Einsatz eines validierten Suizidscreeninginstruments, wie VISCI (Viennese Instrument for Suicidality in Correctional Institutions) oder SIRAS (Scale for Initial Risk Assessment), mit einer zusätzlichen „intuitiv-klinischen Suizidalitätseinschätzung“ [10].

Um Suizidscreenings als einen standardisierten Teil der Routinen in der JVA zu etablieren, sollten diese Vorgänge digitalisiert und eindeutige Verfahrensanweisungen für die Durchführung und Dokumentation etabliert werden. Ein weiterer Vorteil der Digitalisierung ergibt sich durch vorprogrammierte Benachrichtigungen bei fehlenden Eintragungen. Zusätzliche Monitorings innerhalb des Haftverlaufs werden bei gefährdeten Inhaftierten empfohlen, wenn eine erhöhte Basissuizidalität^1^ vorliegt oder sich der psychische Zustand des Inhaftierten stark oder abrupt verschlechtert. Die Screenings sollten von Personen mit entsprechender Schulung durchgeführt werden, um Auslassungsfehler zu minimieren.

Aus der Datenlage ergibt sich bisher keine Notwendigkeit, die Screenings bei allen Inhaftierten regelmäßig zu wiederholen. Aufgrund steigender Belegungszahlen kann ein psychiatrischer Konsiliardienst die Nachfrage nicht mehr decken [26]. Daher sollten Inhaftierten der UHA dauerhaft auch psychiatrisch-psychotherapeutische Maßnahmen zur Verfügung stehen. Deshalb ist zu erwägen, die Facharztstunden in Hamburg – wenn möglich – zu erhöhen, um eine qualifizierte und durchgängige Versorgung sicherzustellen [12]. Zudem ist angesichts der Vielzahl psychisch erkrankter Inhaftierter [27, 28] die Einrichtung einer psychiatrischen Kurzzeitstation vorgesehen, um das Delta zwischen ambulanter und stationärer Versorgung zu verringern (BJV, 2025)^2^.

### Umgang mit soziokulturellen Unterschieden

In Haft kulminieren diverse soziale, kulturelle und gesundheitliche Hintergründe, denen respektvoll begegnet werden muss, wozu gezielte Schulungen notwendig sind. Verbindliche Trainings, die spezifisch auf die Steigerung interkultureller Kompetenz abzielen, sind auf allen Ebenen der interdisziplinären Zusammenarbeit erforderlich, um einer Reproduktion von Rassismus in Haft aktiv entgegenwirken zu können [4, 29]. Weiterführend sollten verpflichtende Schulungen zu gewaltfreier Kommunikation, Deeskalation, psychischen Erkrankungen und selbstschädigendem Verhalten häufiger angeboten und kontrolliert umgesetzt werden [13, 30–32]. Schulungen sollten insbesondere auch über Anzeichen suizidaler Absichten aufklären. Zudem sollte weiterhin proaktiv auf die Sensibilisierung gegenüber Personen der LGBTQIA+ Community, insbesondere im Umgang mit transgeschlechtlichen Personen, hingearbeitet werden, um den praktischen Herausforderungen im Alltag adäquat begegnen zu können [33, 34].

Zur Realisierung der Fort- und Weiterbildung von Mitarbeitenden sollte die Frequenz der Fortbildungsveranstaltungen erhöht werden und das proaktive obligatorische Einplanen der Teilnahme an Fortbildungsveranstaltungen im Dienstplan während der Arbeitszeit sowie regelmäßige Teamtrainings in Kommunikation und Deeskalation verankert werden.

### Strukturelle und bauliche Anpassungen

Der besonders gesicherte Haftraum (bgH) wird von Betroffenen oft als belastend empfunden, daher sollte die Aufenthaltsdauer darin möglichst kurz sein [35, 36]. Bei anhaltender akuter Suizidalität wird statt bgH eine Kombination aus medikamentöser Behandlung (Antidepressivum) und Verlegung in einen „leisen Bereich“ wie die Krankenstation empfohlen [10]. Alternativ kann eine „Notgemeinschaft“ eingerichtet werden, bei der 2 Inhaftierte einen Haftraum teilen oder 2 Räume über einen Transit verbunden werden.

In der JVA München wurde das britische „Listener-Programm“ erprobt und als sinnvoll erachtet. Dieses Programm unterstützt Neuzugänge, indem geschulte Mitinhaftierte als Ansprechpersonen (Listener) in Krisensituationen zur Verfügung stehen [37]. In der ersten Nacht können sie – mit Zustimmung aller Beteiligten – gemeinsam in „Durchbruchszellen“, die miteinander in Verbindung stehen, untergebracht werden. Der Listener kann den Einschluss jederzeit abbrechen.

Außerdem empfiehlt die Nationale Stelle zur Verhütung von Folter (NSVF) seit 2018 statt bgH die Einführung von Suizidpräventionsräumen [38]. In einer Studie aus Leipzig wurde 2023 gezeigt, dass damit die bgH-Nutzung und -Aufenthaltsdauer reduziert werden konnten [39]. Diese Räume sollen sowohl sicher sein als auch mit geeigneten Materialien ausgestattet und durch Sichtfenster oder Kameras überwacht werden und dennoch die Privatsphäre wahren [40]. Sitzgelegenheiten, z. B. Schaumstoffwürfel, verbessern den Aufenthalt [41]. In Hamburg wurden Ryno-Hocker (sicherheitsoptimierte Sitzhocker) im bgH ergänzt. Zudem sollte die Nähe zu einem Dienstzimmer für persönlichen Kontakt gewährleistet sein. Diese Suizidpräventionsräume sind für Personen gedacht, die Suizidgedanken äußern, aber nicht akut suizidal sind.

### SARS-CoV-2-Pandemie

Ein Kausalzusammenhang zwischen pandemiebedingten Einschränkungen und den Suiziden konnte nicht nachgewiesen werden (solche Ereignisse – außerhalb von Haftbedingungen – wurden zu Beginn der Pandemie als „Corona-Suizide“ medial thematisiert; [42]). Die Prävalenzspitzen 2020 und 2021 sowie der Rückgang in den folgenden Jahren deuten möglicherweise darauf hin, dass sich Inhaftierte mit der Zeit an die strengen Regelungen angepasst haben. Insbesondere in den ersten beiden Pandemiejahren waren die Einschränkungen im Justizvollzug gravierend. Einzelunterbringung, Einsamkeit und ausbleibende soziale Kontakte stellten dabei verstärkte Risikofaktoren dar [12]. Internationale Studien zur Auswirkung der Pandemie auf Suizide in Haft sind rar; in der Schweiz wurde ein Anstieg von selbstverletzendem Verhalten und Suizidversuchen in Haft um 57 % im Vergleich zum Vorjahr festgestellt [43]. In Studien von Dahle et al. und Baier et al. wurde ein Anstieg von Suizidgedanken beobachtet, je einschränkender die pandemischen Regeln wahrgenommen wurden [44, 45].

Das Quarantänekonzept in Haftanstalten sollte angepasst werden, um einer zukünftigen Ausnahmesituation wie einer Pandemie gewachsen zu sein. Um dem Verlust sozialer Kontakte entgegenzusteuern, sollten die Telefonkontingente aufgestockt und die Ausgabe von Fernseh- und Videospielgeräten unabhängig vom Vorhandensein eigener finanzieller Mittel ermöglicht werden (wurde in Hamburg umgesetzt). Zudem sollte ein regelmäßiges Umschlussangebot eingeplant werden, um Inhaftierten einen persönlichen Austausch zu ermöglichen. Zusätzlich könnten die durch die Pandemie für Besuchs- und Anwaltsgespräche angeschafften Trennscheiben bspw. in den Gemeinschaftsräumen oder bei Umschluss aufgestellt werden, um den sozialen Kontakt unter den Inhaftierten weiterhin zu ermöglichen. Zudem sollte eine personelle Aufstockung erfolgen, um Ausfälle zu kompensieren und eine kontinuierliche Ansprechbarkeit für Inhaftierte zu gewährleisten.

### Humaner Strafvollzug

Ein humaner Strafvollzug ist die wirksamste Prävention gegen selbstschädigendes Verhalten, suizidale Absichten und weitere negative Haftfolgen. Studien betonen die Bedeutung von Sport- und Gruppenangeboten zum Stressabbau [46–48]. Um diesem Bedarf in Hamburg gerecht zu werden, sollten Kooperationen mit externen Trägern, etwa dem Hamburger Fürsorgeverein, ausgebaut werden. Besonders in den ersten Tagen nach der Zuführung ist eine engmaschige Betreuung der Inhaftierten essenziell, da das Suizidrisiko besonders hoch ist [12, 49]. Zudem hielten sowohl Mitarbeitende als auch Inhaftierte die Möglichkeit zur Kontaktaufnahme mit der telefonischen Seelsorge für hilfreich.

Da Suizide laut Aktenanalyse gehäuft an Samstagen auftreten, sollten am Wochenende verstärkt Freizeitangebote gemacht werden. Besonders auf geschlossenen Stationen sollte sozialer Austausch ermöglicht werden. Opitz-Welke und Konrad betonen, dass suizidale Krisen nicht allein durch klinische Maßnahmen wie Psychotherapie verhindert werden können. Ebenso wichtig ist die Einbindung in alltägliche Abläufe und soziale Kontakte durch Arbeit oder andere Angebote in Haft. Zudem sollte neu zugeführten Inhaftierten unmittelbar der Zugang zu Medien wie Fernsehen, Radio oder Büchern gewährt werden, um Stress abzubauen und die Eingewöhnung zu erleichtern [10].

### Limitationen

Die vorliegende Arbeit ergänzt die bisherigen Erhebungen im Hamburger Strafvollzug und überblickt die Suizidfälle einer Metropolregion sowie die Gedanken und Wünsche von Mitarbeitenden und Inhaftierten aus der Hamburger UHA. Es handelt sich jedoch um lokale Daten und möglicherweise spezifische Verhältnisse, die eine unkritische Generalisierung auf andere Standorte nicht zulassen. Zudem flossen lediglich vollendete Suizidfälle in die quantitative Auswertung ein, da diese durch das besondere Hamburger Leichensystem vollständig erfasst werden konnten. Über etwaige Suizidversuche in den Hamburger Justizvollzugsanstalten liegen keine belastbaren statistischen Maßzahlen vor. Im Rahmen der Interviews können keine Verallgemeinerungen getroffen werden. Kausale Zusammenhänge zwischen den in dieser Studie erwähnten Risikofaktoren aus den quantitativen und qualitativen Auswertungen können aufgrund der niedrigen Fallzahlen und Limitierungen in der statistischen Auswertung nicht hergestellt werden.

## Fazit

Die jährliche Anzahl der verwirklichten Suizide in den Hamburger Haftanstalten hat im Vergleich zu den vorherigen beiden Erhebungszeiträumen absolut und relativ abgenommen. Trotz der Abnahme der Suizide in den Hamburger Haftanstalten sollten aktuelle Entwicklungen wie die Auswirkungen der SARS-CoV-2-Pandemie oder ein kultursensibler Umgang mit Inhaftierten beachtet werden, da sie den Stress der Inhaftierten erhöhen könnten, wodurch die Suizidgefährdung verstärkt werden könnte. Die Ergebnisse unterstreichen die Notwendigkeit, weiterhin systematische, strukturelle und psychologische Maßnahmen zu entwickeln, um Suizidalität im Strafvollzug kontinuierlich zu adressieren und ihr entgegenzuwirken.

## Supplementary Information

Leitfragen mit Folgefragen (Anhänge A und B), weitere Informationen zu den wesentlichen Items (Anhang C)

## Data Availability

Die während der vorliegenden Studie verwendeten Datensätze sind auf begründete Anfrage bei der korrespondierenden Autorin erhältlich.

## Footnotes

1 „Die Basissuizidalität beschreibt das Ausmaß der suizidalen Gefährdung eines Menschen vor dem Hintergrund seiner bisherigen Lebens- und Krankheitsgeschichte sowie seiner aktuellen Situation. Somit drückt die Basissuizidalität das grundsätzliche Risiko einer Person für die Entwicklung von suizidalen Gedanken oder Handlungen aus und ist losgelöst vom aktuellen Zustand der Person zu betrachten. Sie ergibt sich aus dem Vorliegen spezifischer Risikofaktoren“ (Untersuchungshaftanstalt Hamburg Anstaltsverfügung, DG2: Behandlung und Betreuung, SG2/001 V.8, letzte Änderung 10.05.2021).

2 Freie und Hansestadt Hamburg – Behörde für Justiz und Verbraucherschutz (2025, 7. Januar). Justizvollzug richtet psychiatrische Kurzzeitstation ein. Abgerufen am 26.10.2025, von https://www.hamburg.de/politik-und-verwaltung/behoerden/bjv/aktuelles/2025-01-07-bjv-justizvollzug-richtet-psychiatrische-kurzzeitstation-ein-1006338.
