# Supplementary material for: Suicide prevention in Hamburg’s penal system—analysis of cases from 2013–2022 and evaluation of interviews with inmates and staff
Source: Bundesgesundheitsblatt Gesundheitsforschung Gesundheitsschutz. 2025 Nov 12;68(12):1407–15. [Article in German] doi: 10.1007/s00103-025-04154-x (PMC12669344; doi:10.1007/s00103-025-04154-x)
Supplement: Supplementary file 1 — Leitfragen mit Folgefragen (Anhänge A und B), weitere Informationen zu den wesentlichen Items (Anhang C) [file 103_2025_4154_MOESM1_ESM.pdf]

Onlinematerial

Anhang A Leitfadeninterview Inhaftierte

EINSTIEG
Hallo mein Name ist … Ich bin Mitarbeiterin im Projekt „Todesfälle in Haft“ und möchte mich zuerst für Ihre Teilnahme bedanken.<br><br>Soll ich Sie duzen oder siezen?<br><br>Wenn es für Sie in Ordnung ist, würde ich nun gerne das Diktiergerät einschalten und mit unserem Interview beginnen. Bei den Antworten werde ich Sie nicht unterbrechen, sondern erstmal nur zuhören. Lassen Sie sich Zeit. Alles was für Sie wichtig ist, ist für mich interessant.

EINSTIEGSFRAGE		
Zum Anfang würde ich gerne etwas über Ihre Person erfahren. Erzählen Sie mir einfach das, was Ihnen zu sich selbst einfällt.		
**Inhaltliche Aspekte**	**Aufrechterhaltungsfragen**	**Nachfragen**
Leben außerhalb des Vollzugs<br><br>Beziehungen<br><br>Arbeit	- Können Sie mir genauer etwas über … erzählen?<br><br>- Wie haben Sie das empfunden?	- Könnten Sie mir noch etwas zu Ihrer Familie / Arbeit / etc. erzählen?

ÜBERGANG		
Wie haben Sie den U-Haftantritt bzw. den Übergang erlebt?<br><br>Wie waren die ersten Tage?		
**Inhaltliche Aspekte**	**Aufrechterhaltungsfragen**	**Nachfragen**
- Bewertung der Erfahrungen<br><br>- Stress- / Druckmomente	- Können Sie die Situation noch mal genauer schildern?<br><br>- Wie haben Sie sich gefühlt?	- Gab es bei Problemen einen Ansprechpartner?<br><br>- Wenn JA, wer war der Ansprechpartner?<br><br>- Wie lange sind Sie schon hier?

HAFTALLTAG		
Wie sieht Ihr Haftalltag aus?		
**Inhaltliche Aspekte**	**Aufrechterhaltungsfragen**	**Nachfragen**
- Reflektion der U-Haft Zeit	- Können Sie darauf noch mal genauer eingehen?	- Wie erleben Sie die Nächte in der Untersuchungshaft?<br>- Wie erleben Sie die Tage?<br>- Welche Möglichkeiten der Freizeitgestaltung gibt es?

KONTAKT		
a) Wie ist der Kontakt unter den Inhaftierten?<br>b) Wie ist der Kontakt zwischen Inhaftierten und Mitarbeitenden des Vollzugs?<br>c) Wie ist der Kontakt unter den Mitarbeitenden?<br>d) Wie ist der Kontakt zu Ihrer Familie / Freund:innen?		
**Inhaltliche Aspekte**	**Aufrechterhaltungsfragen**	**Nachfragen**
- Reflektion der Hierarchie<br>- Problemwahrnehmung<br>- Informationsfluss	- Was meinen Sie mit …?<br>- (Wie äußert sich das …?)	- Können Sie das Verhalten der Mitarbeitenden nachvollziehen?<br>- Wird eine Hierarchie deutlich?<br>- Wie spiegelt sich das wider?<br>- Haben sich Veränderungen in Bezug auf Besuch seit der Corona-Pandemie ergeben?

AUSSTATTUNG DER EINRICHTUNG I		
Wie beurteilen Sie die Ihnen zur Verfügung stehende Einrichtung allgemein bzw. in Ihrem Haftraum?		
**Inhaltliche Aspekte**	**Aufrechterhaltungsfragen**	**Nachfragen**
- Materielle Ressourcen-Zufriedenheit<br><br>- Räumliche und technische Ressourcen	- Beschreiben Sie bitte einmal Ihren Haftraum.<br><br>Wie wirkt das auf Sie?<br><br>- Was würden Sie allgemein an den Haftbedingungen ändern?	- Wie ist die Einrichtung außerhalb Ihres Haftraums?<br><br>- Was fehlt Ihnen am meisten?<br><br>- Wie empfinden Sie die Einzelhaftunter-bringung?

AUSSTATTUNG DER EINRICHTUNG II		
Was halten Sie davon, Ihre persönlichen Anliegen bei einer geschulten Person der telefonischen Seelsorge anonym mitteilen zu können?		
**Inhaltliche Aspekte**	**Aufrechterhaltungsfragen**	**Nachfragen**
- zusätzliches Angebot	- Wie würden Sie sich mit dem Hilfsangebot fühlen oder damit umgehen?	- Denken Sie, es könnte eine hilfreiche Maßnahme sein, um den Alltag zu verbessern?<br><br>- Würden Sie die Maßnahme selbst in Anspruch nehmen wollen?

Corona-Pandemie und Empfinden		
Inwiefern hat sich Ihr Empfinden in Haft seit der Corona-Pandemie verändert?		
**Inhaltliche Aspekte**	**Aufrechterhaltungsfragen**	**Nachfragen**
- Verschiebung des Verhandlungs- und anderer Termine vor Gericht<br>- Keine Freizeitgruppen oder andere Aktivitäten<br>- Quarantäne und längerer Einschluss<br>- Veränderung, Angstempfinden	- Was meinen Sie mit…?<br>- (Wie äußert sich das…?)	- Inwiefern haben die veränderten Sicherheitsmaßnahmen, bspw. das Tragen einer Maske, Quarantäne und der damit verbundene längere Einschluss einen Einfluss auf Ihre Stimmung?

ERFAHRUNGEN		
Welche außergewöhnlichen negativen Situationen haben Sie bereits in der U-Haft erlebt?		
**Inhaltliche Aspekte**	**Aufrechterhaltungsfragen**	**Nachfragen**
- Grenzerfahrungen in der U-Haft<br>- Erfahrungen mit Suizid!!! *(Frage muss gestellt werden)*<br>- Gefühle / Gedanken bei dem Ereignis	- Beschreiben Sie bitte, wie es Ihnen in der Situation ging?	- Sind Ihnen schon einmal in der U-Haft Suizidgedanken gekommen?<br>- bei Ja: Erzählen sie davon.<br>- bei Nein: Kennen sie jemanden mit Suiziderfahrungen- oder Gedanken? Wie beschrieb die Person das?<br>Gab es andere Vorfälle, die Sie sehr beschäftigt haben?<br>- Gibt es Ihrer Meinung nach vorbeugende Maßnahmen?

AUSSTIEG
Gibt es zum Schluss noch etwas in Bezug auf Suizid in der U-Haft, was sie noch sagen möchten?

DANKE
Vielen Dank für Ihre Zeit und Mühe, die Sie sich genommen haben. Es war sehr interessant Ihnen zu zuhören. Ich wünsche Ihnen alles Gute für die Zukunft und werde Ihnen nun als kleines Dankesschön für Ihre Teilnahme die 5 Euro auf Ihr Konto überweisen.

Anhang B Interviewleitfaden Mitarbeitende

EINSTIEG
Hallo mein Name ist … Ich bin Mitarbeiterin im Projekt „Todesfälle in Haft“ und möchte mich zuerst für Ihre Teilnahme bedanken.<br><br>Wenn es für Sie in Ordnung ist, würde ich nun gerne das Diktiergerät einschalten und mit unserem Interview beginnen. Bei den Antworten werde ich Sie nicht unterbrechen, sondern erstmal nur zuhören. Lassen Sie sich Zeit. Alles was für Sie wichtig ist, ist für mich interessant.

EINSTIEG / ARBEITSALLTAG		
Um Sie am Anfang ein wenig in Ihrer Tätigkeit kennenzulernen, würde ich Sie bitten, uns Ihre Funktion und Ihre tägliche Arbeit zu beschreiben.		
**Inhaltliche Aspekte**	**Aufrechterhaltungsfragen**	**Nachfragen**
- Beschreibung der Tätigkeit	- Wie meinen Sie…?	Wie sind Sie zu dem Beruf gekommen?<br><br>Wo waren Sie vorher tätig?

AUSSTATTUNG DER EINRICHTUNG		
Wie beurteilen Sie die Ihnen zur Verfügung stehende Einrichtung / Technik / Struktur?		
**Inhaltliche Aspekte**	**Aufrechterhaltungsfragen**	**Nachfragen**
- Materielle Ressourcen-Zufriedenheit<br><br>- Räumliche und technische Ressourcen	- Betrifft Sie das persönlich?<br><br>- Wie beurteilen Sie …?	Wenn Sie keinen Beschränkungen unterliegen würden, welche Anschaffungen würden Sie noch machen?<br><br>Was würden Sie in diesem Bereich ändern?

Corona-Pandemie und Empfinden		
Wie schätzen Sie ein, hat sich das Empfinden der Inhaftierten seit der Corona-Pandemie verändert?		
**Inhaltliche Aspekte**	**Aufrechterhaltungsfragen**	**Nachfragen**
- Verschiebung des Verhandlungs- und anderer Termine vor Gericht<br>- Keine Freizeitgruppen oder andere Aktivitäten<br>- Quarantäne und längerer Einschluss<br>- Veränderung Angstempfinden	- Was meinen Sie mit…?<br>- (Wie äußert sich das…?)	- Beschreiben Sie bitte, ob Sie denken, dass sich das Angstempfinden der Inhaftierten seit der Corona-Pandemie verändert hat?

Verhaltensänderung Inhaftierte		
Wie würden Sie das Verhalten von Inhaftierten in den letzten 10 Jahren in Bezug auf eine mögliche extremistische Einstellung beurteilen?		
**Inhaltliche Aspekte**	**Aufrechterhaltungsfragen**	**Nachfragen**
- Verhaltensänderung, die nicht durch Inhaftierung erklärbar war<br>- Ablehnung demokratischer Rechtsordnung	- Was meinen Sie mit…?<br>- (Wie äußert sich das…?)	- Inwiefern sind Ihnen plötzliche Verhaltensänderungen wie Abkehr von bisherigen Verhaltensweisen oder plötzliche Verweigerung bestimmter Regeln aufgefallen?<br>- Konnten Sie eine gewalttätig extremistische Einstellung im Verhalten durch Hinwendung zu einer Gruppierung oder „Glaubensrichtung“ erkennen?

PRAKTISCHE SUIZIDERFAHRUNG		
Welche Erfahrungen haben Sie mit Suizid in der Untersuchungshaft gemacht?		
**Inhaltliche Aspekte**	**Aufrechterhaltungsfragen**	**Nachfragen**
- Suiziderfahrung<br>- Nachbesprechung des Suizids<br>- Umgang mit Suizid in der Untersuchungshaft	- Was ging Ihnen in der Situation durch den Kopf?<br>- Wie haben Sie sich gefühlt?	BEI ERFAHRUNG:<br>Wie würden Sie Ihrer Meinung nach suizidgefährdete Gefangene als solche zu erkennen wissen?<br>Wie wurden Sie vor bzw. nachbereitet?<br><br>KEINE ERFAHRUNG:<br>Stellen Sie sich vor, jemand möchte sich umbringen, wie würden Sie mit der Situation umgehen?

THEORETISCHE ERFAHRUNG		
Welche Erfahrungen haben Sie in Ihrer Ausbildung / Beruf in Bezug auf praktisch Anwendbares für den Umgang mit suizidalen Gefangenen gemacht?		
**Inhaltliche Aspekte**	**Aufrechterhaltungsfragen**	**Nachfragen**
- Lehrmaterialien zu Suizid<br>- Fortbildungen / Konferenz / Tagungen	- Können Sie einmal genauer darauf eingehen, was Sie mit… meinen?	- Welche Erfahrungen haben Sie mit Fortbildungsmaßnahmen in Bezug auf Suizid gemacht?<br>Haben Sie ansonsten etwas darüber erfahren?<br>Welche Präventionsmaßnahmen kennen Sie?

VERBESSERUNGSVORSCHLÄGE		
**Inhaltliche Aspekte**	**Aufrechterhaltungsfragen**	**Nachfragen**
- Wünsche<br>- Ideen<br>- Vorstellungen	- Ist das Ihrer Meinung nach auch umsetzbar?	Gibt es auch interne / externe Verbesserungen?

AUSSTIEG
Gibt es zum Schluss noch etwas in Bezug auf Suizid in der U-Haft, was sie noch sagen möchten?

DANKE
Vielen Dank für Ihre Zeit und Mühe, die Sie sich genommen haben. Es war sehr interessant, Ihnen zu zuhören. Ich wünsche Ihnen alles Gute für die Zukunft und noch einen schönen Tag.

Anhang C: Items der quantitativen Aktenanalyse, auszugsweise

Soziodemografische Daten, Gesundheitsdaten (einschließlich Items zu etwaigen SARS-CoV-2-Erkrankungen und zur Impfung gegen das Virus), Informationen zu sozialen Kontakten, zum aktuellen Tatvorwurf, zur Haftart / zur Vollzugsform zur Aufenthaltsdauer im Gefängnis bis zum Ereignis / zum Vollzugsablauf, zum Suizid und ggf. vorangegangenen Suizidversuchen / zum früheren Suizidrisiko und entsprechenden Sicherheitsmaßnahmen, zu früheren Haftstrafen und derzeit laufenden Verfahren, zum Zugangsgespräch, einschließlich Suizidscreeningbogen, zum Delikt, Suchterkrankungen und aktuellem Verhalten, zu Einschüchterungen und Drohungen in Haft und wie damit umgegangen wurde, zu Abschiedsbriefen und zu Substanzmissbrauch. Außerdem wurden Daten darüber gesammelt, ob die Person während der Inhaftierung beim Psychologischen Dienst vorgestellt wurde und Informationen zu durch SARS-CoV-2-Pandemie verursachten Anpassungen und damit verbundenen Veränderungen des Sicherheitsgefühls vorlagen.
